# A guardian angel: how immune factor RPS2 is activated by pathogen effector AvrRpt2

**DOI:** 10.1093/plcell/koag226

**Published:** 2026-07-29

**Authors:** Annabelle Campbell

**Affiliations:** Assistant Features Editor, The Plant Cell, American Society of Plant Biologists, United States; Department of Biology, Duke University, Durham, North Carolina

For a plant's immune system to function properly, it must specifically detect and robustly react to pathogen infection. Bacterial pathogens such as *Pseudomonas syringae* use the type 3 secretion system, a needle-like structure to inject effector proteins into plant cells, which promote susceptibility by suppressing host immunity and creating a suitable niche (Wang et al. 2022). In return, plants have evolved NLRs (named for their Nucleotide-binding and Leucine-rich Repeat domains), immune receptors that specifically recognize effector proteins and subsequently trigger host defense, often including a type of cell death called the hypersensitive response (HR) (Jones and Dangl 2006).

Some NLRs have evolved to detect the activity of an effector rather than an epitope within it. In this “guarding” system, the NLR (or guard) recognizes an effector-induced modification of a host protein (or guardee) (Dangl and Jones 2001). One well-studied example of such guarding is seen with the *Arabidopsis thaliana* RPM1-interacting protein 4 (RIN4), which is a small, unstructured protein that is C-terminally anchored to the plasma membrane (PM) where it negatively regulates plant immunity (Sun et al. 2014). Multiple *P. syringae* effectors modify RIN4 to enhance its defense suppressive activity (Toruño et al. 2019). However, these modifications also activate cognate guard NLRs that promote immunity.

AvrRpt2 is a *P. syringae* effector protease that cleaves RIN4 into a membrane-tethered (MT) cleavage product, AvrRpt2-cleavage product 3 (ACP3), and a non–membrane-tethered (NMT) cleavage product, ACP2, and subsequently activates the guard NLR RPS2 (Mackey et al. 2003). Interestingly, RPS2 activation upon RIN4 proteolysis by AvrRpt2 requires the presence of the RIN4-interacting protein NON-RACE-SPECIFIC DISEASE RESISTANCE1 (NDR1). In other words, RIN4 proteolysis alone is not sufficient for RPS2 activation. In recent work, Ahmed Afzal and colleagues (Afzal et al. 2026) explore how the interplay between RIN4 cleavage products and NDR1 activates RPS2.

In its native state, MT RIN4 suppresses RPS2 activity. To test the effect of untethering RIN4, the authors expressed full-length or near full-length NMT RIN4 derivatives (not the AvrRpt2 cleavage products) in *A. thaliana*. Interestingly, these NMT derivatives caused an HR dependent on RPS2. These data indicate that NMT RIN4 (not the elimination of RIN4 by AvrRpt2) drives RPS2 activation.

As previously mentioned, NDR1, a GPI-anchored protein localized to PM, is required for AvrRpt2-mediated activation of RPS2. The authors found that in the absence of NDR1, the NMT RIN4 derivatives were also unable to activate RPS2, further reinforcing NDR1's role in the activation of RPS2 at its native location, the PM.

Because the NMT RIN4 derivatives are soluble, the authors sought to determine where RPS2 is activated. NMT RIN4 was modified with either a nuclear export signal (NES) or nuclear localization signal (NLS). Consequently, RPS2 activation was observed only in plants expressing NES-NMT RIN4, indicating that RIN4 mediates RPS2 activation in the cytosol. TurboID proximity labeling was then used to confirm that RPS2 is closely associated with both MT and NMT RIN4. Interestingly, coexpression of NMT RIN4 greatly diminished labeling of MT RIN4 by RPS2, suggesting that NMT RIN4 outcompetes MT RIN4 for association with RPS2 at the PM.

Based on these data, the authors formulated a model (see Fig. 1): under normal conditions, RIN4 and NDR1 suppress RPS2 at the PM. Upon *P. syringae* infection, the pathogen effector AvrRpt2 cleaves RIN4 into NMT ACP2 and MT ACP3, which remains associated with NDR1 and RPS2. ACP2 is then recruited to RPS2 by NDR1, where it outcompetes ACP3 and activates RPS2 to trigger cell death. Errant activation of RPS2 is detrimental to the plant's health, and this system keeps its activity tightly guarded yet still rapidly responsive to cleavage of RIN4 by AvrRpt2.

**Figure 1:**
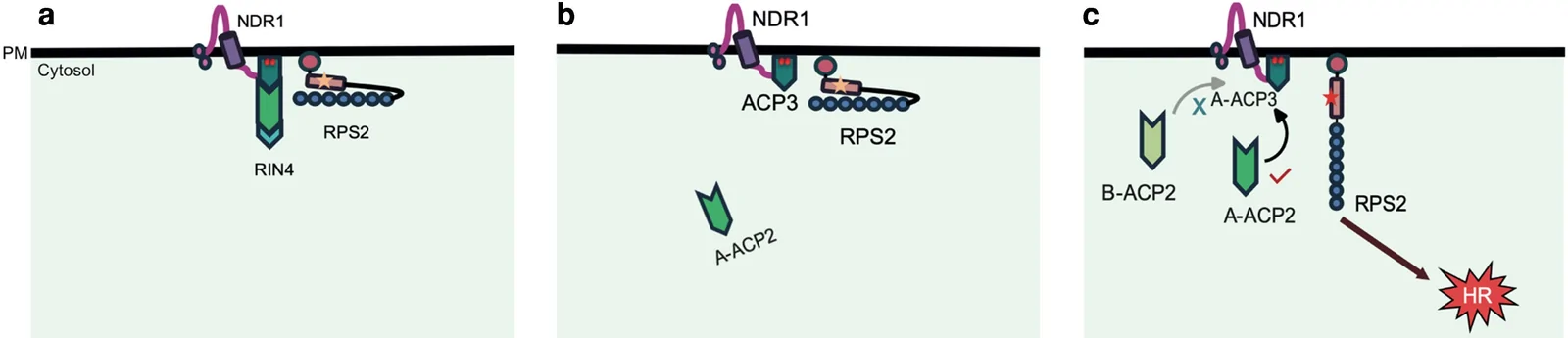
Model for RPS2 activation by cleaved RIN4 products. **a)** RIN4 and NDR1 suppress RPS2 at PM. **b)** After cleavage by AvrRpt2, MT derivative ACP3 continues to suppress RPS2. **c)** NMT derivative ACP2 outcompetes ACP3 and activates RPS2 to promote HR. A-ACP2 and A-ACP3 are derived from the same species' RIN4, B-ACP2 is derived from a different species' RIN4. B-ACP2 cannot activate A-ACP3-suppressed RPS2, representing interspecies incompatibility. Adapted from Afzal et al. 2026.

Importantly, this mechanism is widespread: pathogen homologs of AvrRpt2 target RIN4 homologs for proteolysis in crops such as soybean and potato, which are then recognized by guard NLRs like in *Arabidopsis*. Interestingly, the cleavage products of RIN4 homologs do not show interspecies compatibility; in other words, one species' ACP2 cannot activate RPS2 suppressed by another species' ACP3 (see Fig. 1). Thus, these interactions have been maintained over long evolutionary timescales, further emphasizing the utility of this guarding mechanism.

This study contributes to a growing body of work characterizing the complex molecular mechanisms involved in the interactions of guarding NLRs, host guardees, and pathogen effectors. Continued research into plant immune systems and plant/pathogen relationships is an important step for engineering more resilient plants, especially in the face of a changing climate and growing population.

## Recent related articles in *The Plant Cell*

- Wang et al. (2026) showed that IPA1 and OsNPR1 physically interact in the nucleus to enhance the transcriptional activity of IPA1 in rice immunity and stabilize OsNPR1 by protecting it from OsCUL3a-mediated degradation.
- Sun et al. (2024) showed that alternative splicing of the potato resistance gene *RB* balances plant growth and immunity, and the pathogen effector IPI-O1 interacts with the spliceosome to mediate the *RB* splicing and activate immunity.
- Zhong et al. (2026) demonstrated that the transcription factor MYC2 is phospho-regulated by the phosphatase PP2A Bɑ and the kinase EDR1 to mediate powdery mildew resistance.

## Data Availability

The data underlying this article are available at https://doi.org/10.1093/plcell/koag204.
